# Cognitive tasks propagate the neural entrainment in response to a visual 40 Hz stimulation in humans

**DOI:** 10.3389/fnagi.2022.1010765

**Published:** 2022-10-06

**Authors:** Elvira Khachatryan, Benjamin Wittevrongel, Mariska Reinartz, Ine Dauwe, Evelien Carrette, Alfred Meurs, Dirk Van Roost, Paul Boon, Marc M. Van Hulle

**Affiliations:** ^1^Department of Neurology, Ghent University Hospital, Ghent, Belgium; ^2^Department of Neurology, General Hospital Maria Middelares, Ghent, Belgium; ^3^Department of Neuroscience, Laboratory for Neuro- and Psychophysiology, KU Leuven, Leuven, Belgium; ^4^Leuven Brain Institute (LBI), Leuven, Belgium; ^5^Department of Neuroscience, Laboratory for Cognitive Neurology, KU Leuven, Leuven, Belgium; ^6^Department of Neurosurgery, Ghent University Hospital, Ghent, Belgium

**Keywords:** electroencephalography (EEG), electrocorticography (ECoG), Gamma ENtrainment Using Sensory stimulation (GENUS), cognitive decline, cognitive task

## Abstract

**Introduction:**

Alzheimer's disease is one of the great challenges in the coming decades, and despite great efforts, a widely effective disease-modifying therapy in humans remains elusive. One particular promising non-pharmacological therapy that has received increased attention in recent years is based on the Gamma ENtrainment Using Sensory stimulation (GENUS), a high-frequency neural response elicited by a visual and/or auditory stimulus at 40 Hz. While this has shown to be effective in animal models, studies on human participants have reported varying success. The current work hypothesizes that the varying success in humans is due to differences in cognitive workload during the GENUS sessions.

**Methods:**

We recruited a cohort of 15 participants who underwent a scalp-EEG recording as well as one epilepsy patient who was implanted with 50 subdural surface electrodes over temporo-occipital and temporo-basal cortex and 14 depth contacts that targeted the hippocampus and insula. All participants completed several GENUS sessions, in each of which a different cognitive task was performed.

**Results:**

We found that the inclusion of a cognitive task during the GENUS session not only has a positive effect on the strength and extent of the gamma entrainment, but also promotes the propagation of gamma entrainment to additional neural areas including deep ones such as hippocampus which were not recruited when no cognitive task was required from the participants. The latter is of particular interest given that the hippocampal complex is considered to be one of the primary targets for AD therapies.

**Discussion:**

This work introduces a possible improvement strategy for GENUS therapy that might contribute to increasing the efficacy of the therapy or shortening the time needed for the positive outcome.

## 1. Introduction

Alzheimer disease's (AD) is a progressive, life-shortening condition characterized by a gradual decline in cognitive abilities, eventually leading to dementia (Breijyeh and Karaman, 2020; Knopman et al., 2021). The risk for AD-development dramatically increases with age as, according to the Alzheimer's Association report, more than 30% of the population aged above 85 live with AD (Hebert et al., 2013; Association, 2020). Given the globally aging population and the suffering that the disease causes the patients and their caregivers, the social and economic burden associated with AD will progressively grow.

Despite considerable efforts, research toward therapeutic disease-modifying strategies for AD have, so far, not been widely successful. The vast majority of these efforts is built upon the amyloid hypothesis which states that the disease evolves as a result of amyloid accumulation and, subsequent accumulation of more non-specific proteins, such as total and phosphorylated tau, that cause neural injury resulting in neurodegeneration and cognitive decline (Sperling et al., 2011; Jack et al., 2018). Several pharmaceutical therapies aimed at improving amyloid clearance have been developed but reported little success (Breijyeh and Karaman, 2020). Also, Aducanumab, the first ever FDA-approved disease-modifying therapy for AD, despite showing great promise, yields contradictory results (Mullard, 2021).

In recent years, a new therapeutic technique has been described based on “Gamma ENtrainment Using Sensory stimuli” (GENUS; Iaccarino et al., 2016; Adaikkan and Tsai, 2020). This technique presents the subject with sensory (visual and/or auditory) stimulation at a frequency of 40 Hz and elicits neural gamma activations. It has shown promising outcomes in mouse models of AD, where reduced amyloid production and improved clearance in hippocampus and prefrontal cortex, as well as improved recognition and visuospatial memory (Adaikkan et al., 2019; Martorell et al., 2019), have been described following a GENUS “therapy” of 1 h daily sessions over the course of one to several weeks.

According to the *in vivo* studies GENUS stimulation activates microglia, down-regulates the expression of inflammatory proteins therefore having an anti-inflammatory effect as well as modulates synaptic plasticity (Guan et al., 2022). Visual 40 hz stimulation alleviates the cell loss in V1, CA1 cingulate cortex and somatosensory cortex in mice AD-model. TMS with 40 Hz shown to enhance gamma band power in a number of areas including left parietotemporal cortex and in this way improves condition and executive function in AD patients. It was shown also improves connectivity between posterior cingulate cortex and precuneus (Adaikkan and Tsai, 2020; Guan et al., 2022).

However, GENUS studies in humans have, so far, not shown conclusive results (Ismail et al., 2018; Sharpe et al., 2020). While some studies have described improved cognition with a decline in cortical atrophy and strengthening of the default mode network in patients with mild-AD (Chan et al., 2021), and an improved mood and memory in healthy participants (Sharpe et al., 2020), others were unable to observe any change in amyloid accumulation after a 10-day therapy (Ismail et al., 2018).

It is currently unclear why the outcomes of gamma entrainment therapy in humans are inconclusive. The authors of previously mentioned negative report (Ismail et al., 2018), which used 10-day therapies of visual entrainment, suggested that a longer therapy would be required for positive outcomes. While successful reports indeed adapted longer therapies [4 weeks (Sharpe et al., 2020) to 3 months (Chan et al., 2021)], the entrainment-evoking stimulation also differed across the studies [visual (Park et al., 2020), auditory (Sharpe et al., 2020), or audiovisual (Chan et al., 2021)], rendering a direct comparison not trivial.

Another suggestion relates to the activities performed during the therapy. It has been recently shown in mice (Park et al., 2020) that physical exercise during the gamma entrainment therapy (over the course of 4 weeks) reduces amyloid and tau-levels to a greater degree compared to visual stimulation or exercise alone, and in this way improves spatial learning, working and long-term memory. In previous human studies, the activities of the patients enrolled in the therapies have not been well-described. EEG in response to 40 Hz stimulation was also not recorded in all studies (for example Ismail et al., 2018; Sharpe et al., 2020; Chan et al., 2021) making it in some cases difficult to assure that the entrainment actually occurred. These factors render it hard to distill a clear conclusion about the effect of cognitive or physical activity during the entrainment therapy.

With the current study, we aim to investigate the latter issue and deepen our understanding of the exact conditions required for improved gamma entrainment. To this end, we adopted a visual gamma entrainment paradigm (i.e., a 40 Hz flickering stimulation) and combined it with simple visual and non-visual tasks. We captured the evoked entrainment and its spreading in the brain using scalp- and intracranial electroencephalography (EEG) in young cognitively-healthy participants.

Based on previous frequency tagging studies (Silberstein et al., 2001; Toffanin et al., 2009), we hypothesize that the combination of 40 Hz flickering with a cognitive task would evoke a stronger and more widespread entrainment compared to a non-task setting. This cortical spreading is important as traditional visual steady-state responses are believed to be limited to the early visual cortices (Wittevrongel et al., 2018), while entrainment therapies mostly target hippocampal amyloid buildup. Furthermore, given that the visual task requires an interaction with the stimulation paradigm, we hypothesize that it will evoke a stronger (and/or more widespread) entrainment compared to a non-visual cognitive task of similar complexity.

In case our hypothesis holds, and addition of a simple cognitive task during entrainment therapy contributes to its spreading across the cortex or its strengthening, it might open the door for more efficient non-pharmacological and non-invasive gamma entrainment therapies for Alzheimer's disease in humans.

## 2. Results

**Figure 1:**
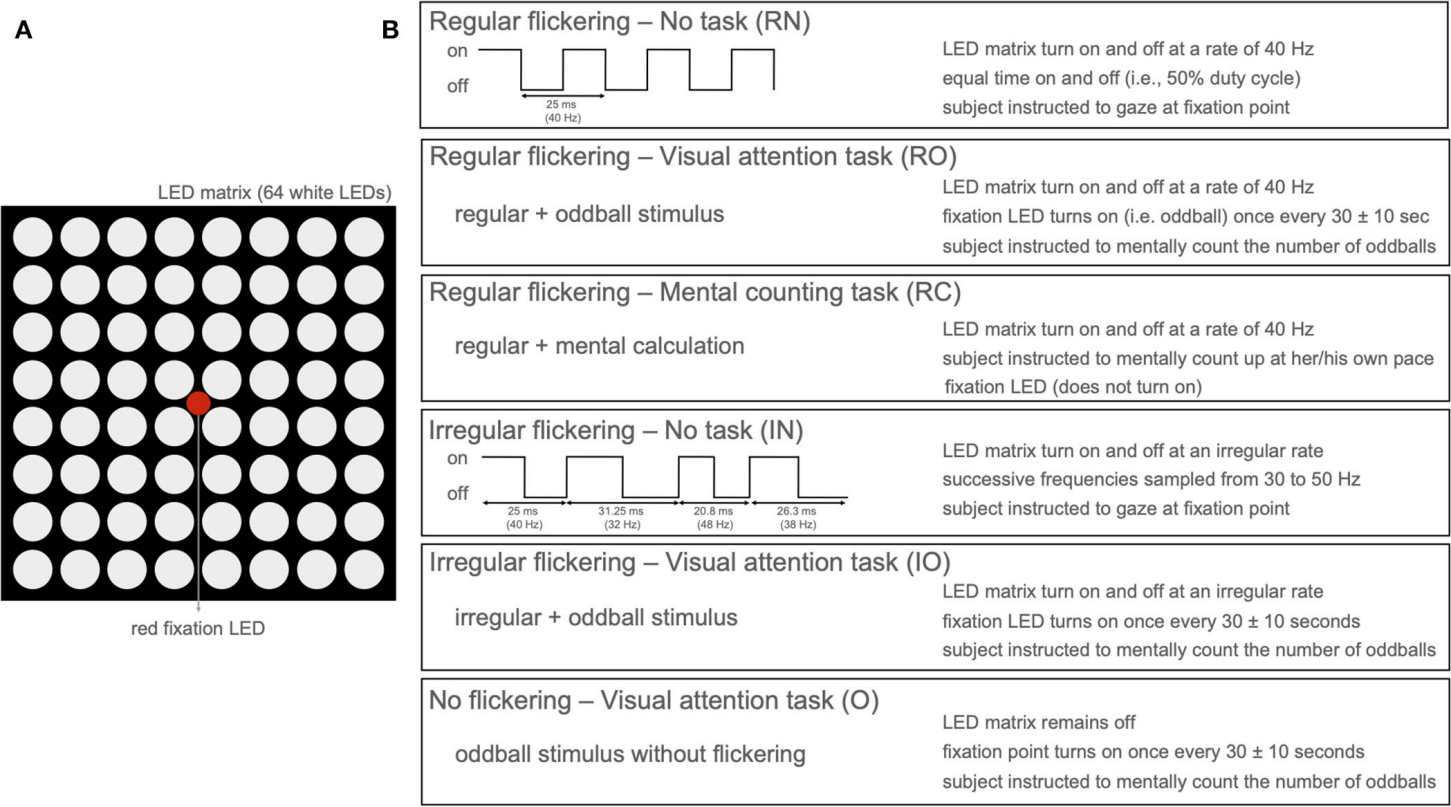
**(A)** LED arrangement of the stimulation device with 8 × 8 LED matrix with a single red fixation LED in the center. **(B)** Stimulation paradigm per condition presenting five conditions with active flickering stimulation (LED matrix on) and one condition with only oddball paradigm without flickering stimulation (only red LED active).

To investigate the effect of cognitive tasks on the gamma entrainment, we developed a paradigm that presents a visual flicker to 16 participants while we recorded their neural responses using scalp-EEG (15 participants) or invasive-EEG (1 participant). The eye movements and fixation was recorded using eye-tracking. Each of the participants completed four sessions each 5 min long, during each of which a visual flickering was presented (Figure 1): 3 sessions of 40 Hz regular [R] flickering and 1 session of irregular [I] flickering using concatenated single periods of frequencies randomly sampled between 30 and 50 Hz. Two out of three R-sessions were combined with a cognitive task—one with mental counting [RC] and one with visual attention (oddball paradigm) [RO]. The remaining R- and I-conditions were coupled with a resting state, i.e., no-task conditions (RN and IN). In addition to these conditions, the 15 participants that underwent scalp-EEG recordings (62 active Ag/AgCl electrodes) were also presented with an irregular flickering combined with a visual attention task (IO condition), and 14 of these participants also completed an attention task without visual flicker [O]. For a detailed description of the experimental paradigm and the analysis methods, we refer the reader to the Section 4.

### 2.1. Cognitive tasks strengthen the gamma entrainment and contribute to its cortical spreading

#### 2.1.1. Eye-tracking

The eyetracking results showed that subjects were continuously attending the stimulation device during all stimulation conditions with the average deviation of 1.7 ± 0.17° in horizontal axis and 1.3 ± 0.47° in vertical axis, while the stimulation device spanned 8.3° visual angle.

#### 2.1.2. Scalp EEG

**Figure 2:**
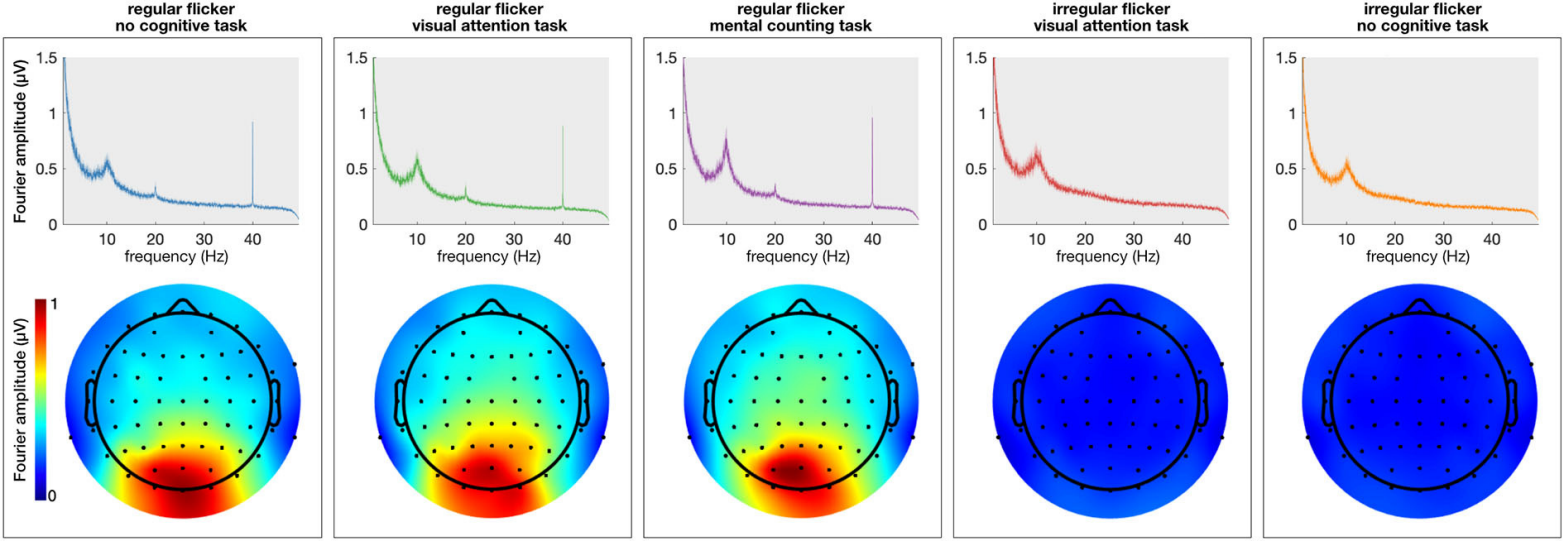
Average (across subjects) frequency spectrum showing gamma entrainment for each of every five stimulation conditions. The **(top)** panel shows the amplitude of the 40 Hz frequency in the frequency spectrum presented in μV and the **(bottom)** panels show the spatial distribution of 40 Hz across the scalp. As one can see, unlike regular stimulation conditions, there is no increase in 40 Hz amplitude in irregular stimulation conditions. As to spatial distribution of the entrainment, as expected, it was the most prominent in the occipital area of the scalp for all regular conditions.

First, the spectral content of the neural responses was obtained using the Fourier transform (frequency resolution of 0.033 Hz). All conditions in which a regular visual flicker was presented exhibit a clear peak at 40 Hz in the spectrum (i.e., the gamma entrainment) while the irregular conditions do not (Figure 2). Noteworthy, the irregular condition that was paired with a task, similar to the irregular-no task condition, did not evoke a change in the 40 Hz amplitude. This suggests that the task *per se* is not responsible for a change in the frequency-of-interest. Given these negative results and in order to keep the message clear, we will discuss only the results on the regular flickering in the remainder of this manuscript. Supplementary Figure S1 further shows the time-frequency spectrum on two representative electrodes, clearly showing the effect of the stimulation on the recorded signals.

**Figure 3:**
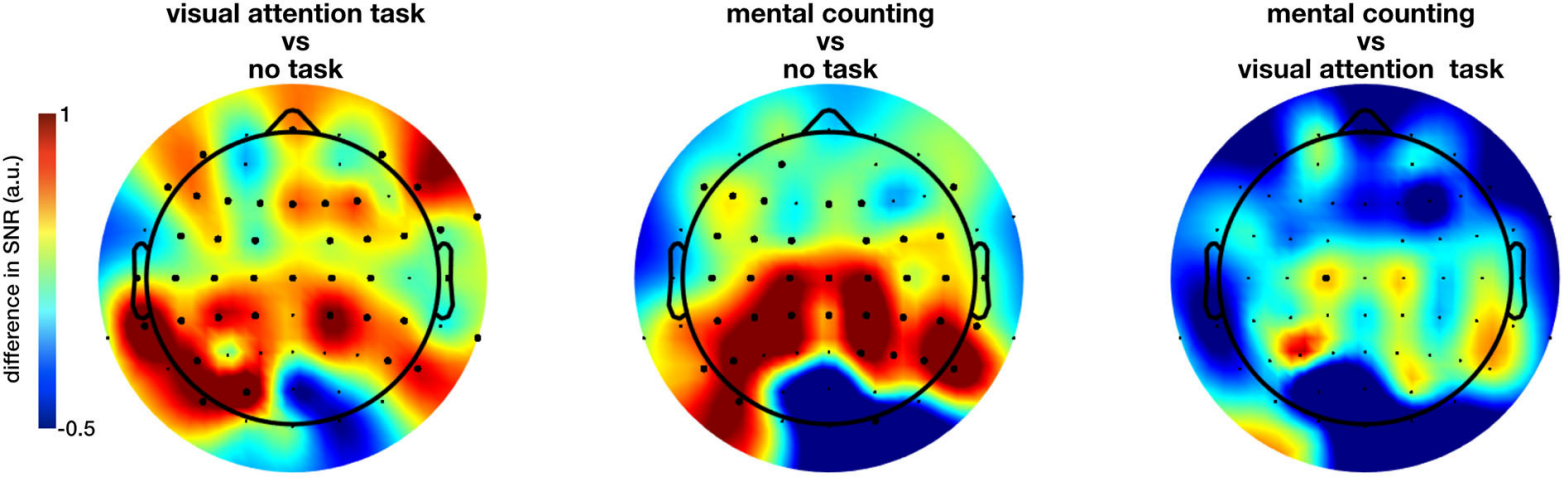
Difference in the gamma entrainment signal-to-noise ratios (ΔSNR) of regular-flickering conditions across subjects. The electrodes at which a statistical significance between conditions is detected are marked with thick dots. The significant difference between conditions with and without cognitive task are always in the anterior regions with visual task being more spread to the centro-frontal areas and non-visual task to the parietocentral regions. Furthermore, even though not statistically significant, one can see that the no-task condition consistently evokes stronger entrainment in the occipital regions in comparison with task conditions.

The extent of the entrainment elicited by the different conditions is further quantified using the signal-to-noise ratio (SNR), which represents the prominence of the 40 Hz amplitude response with respect to the surrounding frequencies (i.e., baseline). Figure 3 shows the difference in scalp topography between each pair of the regular conditions averaged across subjects, effectively showing the spreading of the gamma entrainment over the scalp. Note that electrode with a significant difference are indicates with thicker dots. As quantitative illustration, Table 1 lists the median SNRs and their 95% confidence intervals (CI) for each stimulation condition across all subjects on electrodes O2 and Cz.

**Table 1:** Median SNR and 95% confidence interval for each stimulation condition at two representative electrodes.

	**Regular flickering**			**Irregular flickering**	
	**No task**	**Visual attention task**	**Mental counting task**	**No task**	**Visual attention task**
**O2**	7.55 (6.77–8.48)	7.04 (6.04–7.96)	5.96 (4.98–6.77)	0.95 (0.9–1.04)	1 (0.9–1.07)
**Cz**	3.1 (2.88–3.43)	3.68 (3.38–3.95)	3.96 (3.73–4.26)	0.96 (0.85–1.03)	0.88 (0.79–0.93)

The non-parametric Kruskal-Wallis test shows a significant effect of stimulation condition (RN, RO, RC, IN, and IO) on the SNR at nearly all electrode sites (e.g., on electrode O2, χ^2^ = 810.14, *p* < 0.0001; on electrode Cz, χ^2^ = 736.92, *p* < 0.0001 and on electrode Fz, χ^2^ = 480.96, *p* < 0.0001). When comparing only the three regular conditions using the same method, a large number of electrodes showed a significant effect of cognitive task on the measured SNR, this time mainly at parieto-central and centro-frontal locations. Further pairwise comparison using the Kruskal-Wallis test with FDR correction for *p*-values showed a significant difference between mainly both task conditions and no-task condition (all *p* < 0.05, Figure 3 for the spatial distribution of the difference). Figure 3 suggests that the inclusion of a cognitive task during the sensory stimulus (conditions RC and RO) spreads the effect of the gamma entrainment over a larger cortical area, as nearly all non-occipital scalp electrodes exhibit a significant increase in SNR compared to the non-task (RN) setting (e.g., for electrode CP1, χ^2^ = 12.37, *p* = 0.0004 between RO and RN and, χ^2^ = 16.62, *p* = 0.000046 between RC and RN conditions). In contrary to what was expected, the difference between the two task conditions (i.e., RO and RC) was considerably less pronounced and limited to only electrode C1.

**Figure 4:**
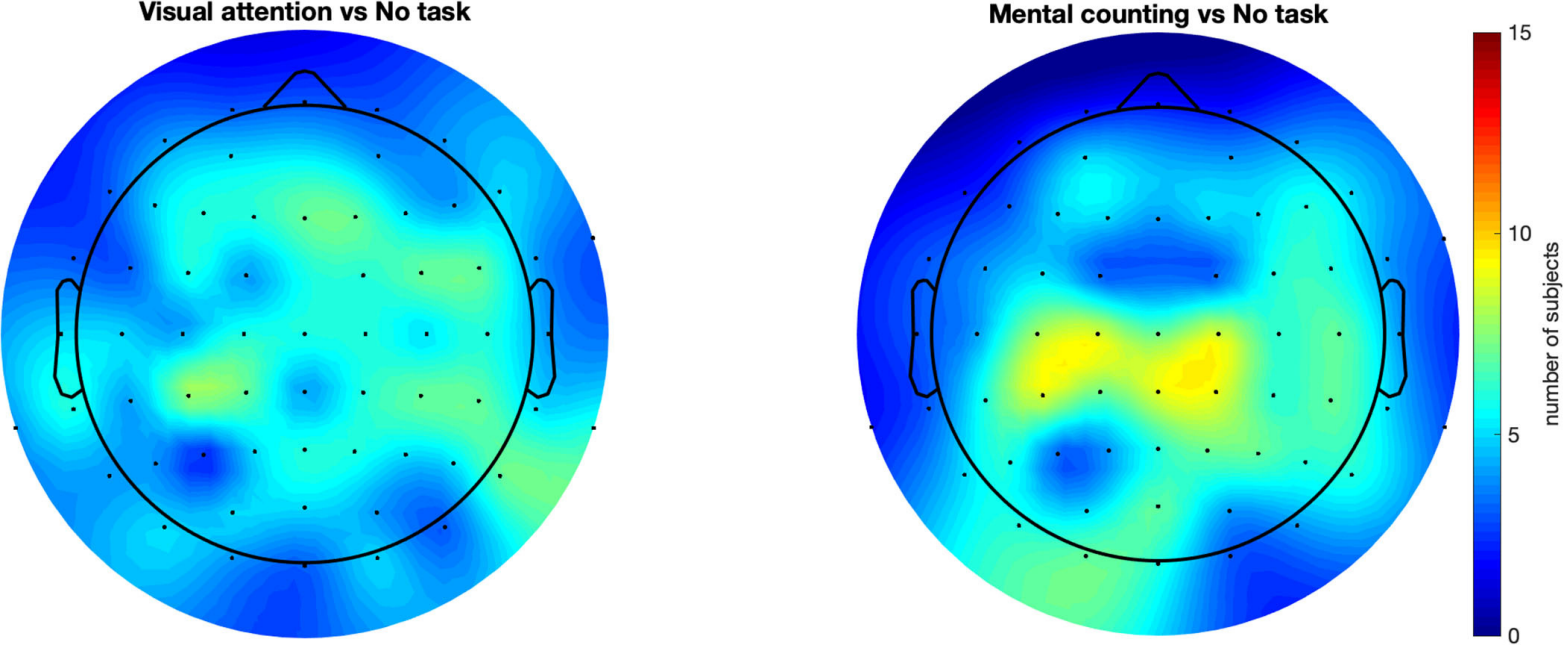
Scalp plots representing for how many subjects each electrode was included in a statistically significant cluster. All compared conditions had regular flickering. This figure suggests a consistency in the results across subjects, showing that majority of the subjects had an anterior propagation of the entrainment with introducing a cognitive task. These results also strengthen the observation in Figure 3 (the spatial distribution for task-conditions), but in this case in a single subject case basis.

In addition to the previous group-level analysis, we further investigated our data on a single-subject level. We adopted the cluster-based permutation test (Maris and Oostenveld, 2007) to statistically quantify the spreading of the gamma entrainment (reflected in an increased SNR compared to the non-task setting) for each subject individually. This method considers not only the temporal properties of EEG signal but also the spatial distribution of the effect and does so using a data-driven approach. This allows us to perform a rigorous statistical comparison while simultaneously preserving the power of our study. The irregular conditions did not show a significantly higher gamma entrainment in any of the subjects. This is expected as these conditions do not satisfy the stationarity criteria to elicit a steady-state neural response. To further corroborate, we compared the two irregular conditions and again obtained practically no significant clusters. When comparing the regular no-task (RN) and visual attention (RO) conditions, 12 out of 15 subjects had at least one statistically significant cluster (median *p* = 0.0005) exhibiting a higher SNR for the RO condition with an average number of 25.5 electrodes per cluster (ranging between 4 and 48 electrodes). This suggests a more widespread effect of the gamma entrainment with the addition of a visual attention task. Similarly, when comparing the mental counting (RC) and no-task (RN) conditions, 10 out of 15 subjects exhibited at least one significant cluster (median *p* = 0.0005) of wider/stronger SNR for the mental counting task, with an average of 26.7 electrodes per cluster (ranging between 3 and 49 electrodes). Finally, when comparing the RO and RC conditions, 12 out of 15 subjects presented clusters (median *p* = 0.0047) where the activation for RC was higher than for RO, with on average 14.75 electrodes per cluster ranging between 4 and 36 electrodes. The difference between the two cognitive task conditions seems to suggest that the topography of the spreading depends on the type of task instructed to the participants. Figure 4 summarizes these results by showing the number of subjects that exhibited a significant effect for each scalp electrode (i.e., for how many subjects that each electrode was included in a significant cluster). This figure shows that, while there is a considerable degree of variability in spreading across subjects, a general trend toward an anterior inclusion is clearly noticeable. Furthermore, it becomes clear that the mental counting task seems more restricted to the central scalp area while the visual attention task also involves the frontal areas.

### 2.2. Cognitive tasks propagate the gamma entrainment to the hippocampus and insula

#### 2.2.1. Intracranial EEG

**Figure 5:**
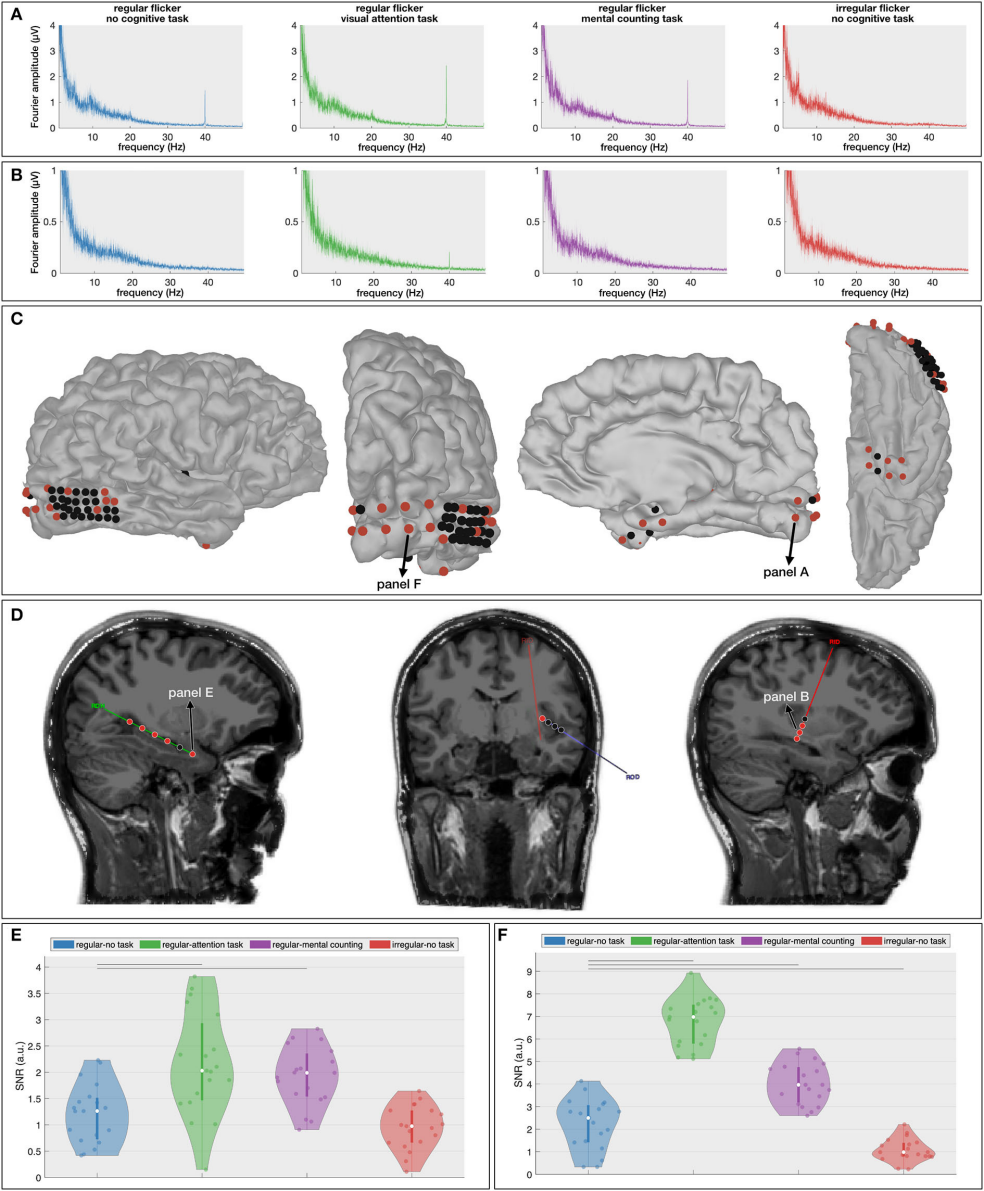
Intracranial results. **(A,B)** Frequency spectra for the four presented conditions at one cortical [**(A)**, primary visual cortex] and one depth [**(B)**, insular cortex] contact. These spectra show the increased gamma entrainment when the subject is instructed to perform a cognitive task during the visual stimulation. **(C,D)** Localization of the cortical **(C)** and depth **(D)** contacts. Electrodes that exhibit a significant difference in gamma entrainment across the four conditions using Kruskall-Wallis test are indicated in red. **(D,E)** Signal-to-noise ratio (SNR) boxplots in response to the four stimulation conditions for one hippocampal contact **(E)** and one occipital electrode **(F)**. Note that their locations are marked in **(C,D)**. The horizontal lines above the boxplots indicate the pairs that exhibit a statistically significant difference with no-task condition (Wilcoxon rank-sum test with FDR correction).

While the results of the scalp-EEG cohort are promising, the evidence is indirect as scalp signals lack the spatial fidelity to make strong claims about cortical involvements. In an effort to corroborate the propagation of the gamma entrainment through the brain with the addition of a cognitive task, we recruited one epilepsy patient who was temporarily implanted with subdural and depth electrodes (63 contacts in total, Figures 5C,D) and repeated the same experiment, albeit with a reduced number of conditions (RN, RO, RC, and IN) in order to adhere to the clinical constraints (see Section 4 for a detailed description). Similar to the scalp-EEG cohort, we extracted frequency spectrum for each condition (frequency resolution of 0.033 Hz; see Figures 5A,B for example spectra at two locations) and calculated the SNR of the 40 Hz component (i.e., the gamma entrainment) for each electrode individually.

The Kruskal-Wallis test revealed a significant effect of stimulation condition on gamma entrainment (Figures 5C,D) on five out of six electrodes implanted in the right hippocampus (e.g., for electrode RHD1, *p* < 0.00001), in four out of eight insular (ROD and RID) electrodes (e.g., on electrode RID1, *p* = 0.00017), in 6 out of 32 electrodes implanted over the right posterior temporal lobe (e.g., on electrode RTG8, *p* = 0.004), in 9 out of 10 electrodes implanted in the right occipital cortex (e.g., on electrode RGO1, *p* < 0.00001) and in six out of eight electrodes implanted in the right anterior lateral and basal temporal cortex (e.g., on electrode RSA4, *p* = 0.0006). This suggests that multiple brain areas, also outside the visual cortex, exhibit gamma entrainment when stimulated visually.

Further multiple comparison with the Wilcoxon rank-sum test (Supplementary Figure S2) and with FDR-correction shows a significant difference in the extent of gamma entrainment between the no-task and cognitive task conditions at hippocampal electrode RHD1 (*p* = 0.0026 between RN and RO and, *p* = 0.003 between RN and RC; Figure 5E). More importantly, no significant difference between regular and irregular no-task conditions was found at this electrode (*p* = 0.27), and the median SNR of both conditions is close to 1 suggesting that the visual stimulation alone has no effect on these neural areas. In contrast, the most superficial contact on the hippocampal probe (RHD6), which is not located within the hippocampal structure, does show a significant effect of gamma stimulation (*p* = 0.0006 between RN and IN conditions) but does not show a difference between regular conditions with and without a cognitive task (*p* = 0.25 between RN and RO; *p* = 0.84 between RN and RC and; *p* = 0.16 between RO and RC, analysis is not shown). For the insular electrodes, the main significant difference is observed between the regular no-task (RN) and visual attention (RO) conditions (e.g., for electrode RID2, *p* < 0.00001) and between the visual attention (RO) and mental counting (RC) conditions (e.g., for electrode RID1, *p* = 0.016). The right posterior temporal lobe is barely involved in the entrainment, while the majority of electrodes implanted over the right occipital cortex (Figure 5F) show significant differences across the stimulation conditions. At the latter locations, in addition to the expected absence of gamma entrainment for the irregular visual flickering (i.e., SNR close to 1), a significantly stronger gamma entrainment is observed for both cognitive task conditions compared to the no-task condition (e.g., *p* < 0.00001 on electrode RGO2, and, *p* < 0.00005 on electrode RGO4 for RO and RC conditions, respectively) and between two conditions with a cognitive task (e.g., on electrode RGO4, *p* < 0.00001 between RO and RC). On the right anterior temporal cortex, the main significant difference is noted between the no-task (RN) and both cognitive task conditions (e.g., on electrode RSA4, *p* = 0.0015 and *p* = 0.0036, for visual attention and for mental counting, respectively). Our results show that different brain areas react differently to the 40Hz flickering stimulation and the presence of a cognitive task influences the propagation of the entrainment.

### 2.3. Unlike regular flickering, the irregular flickering negatively influences the attention processing

**Figure 6:**
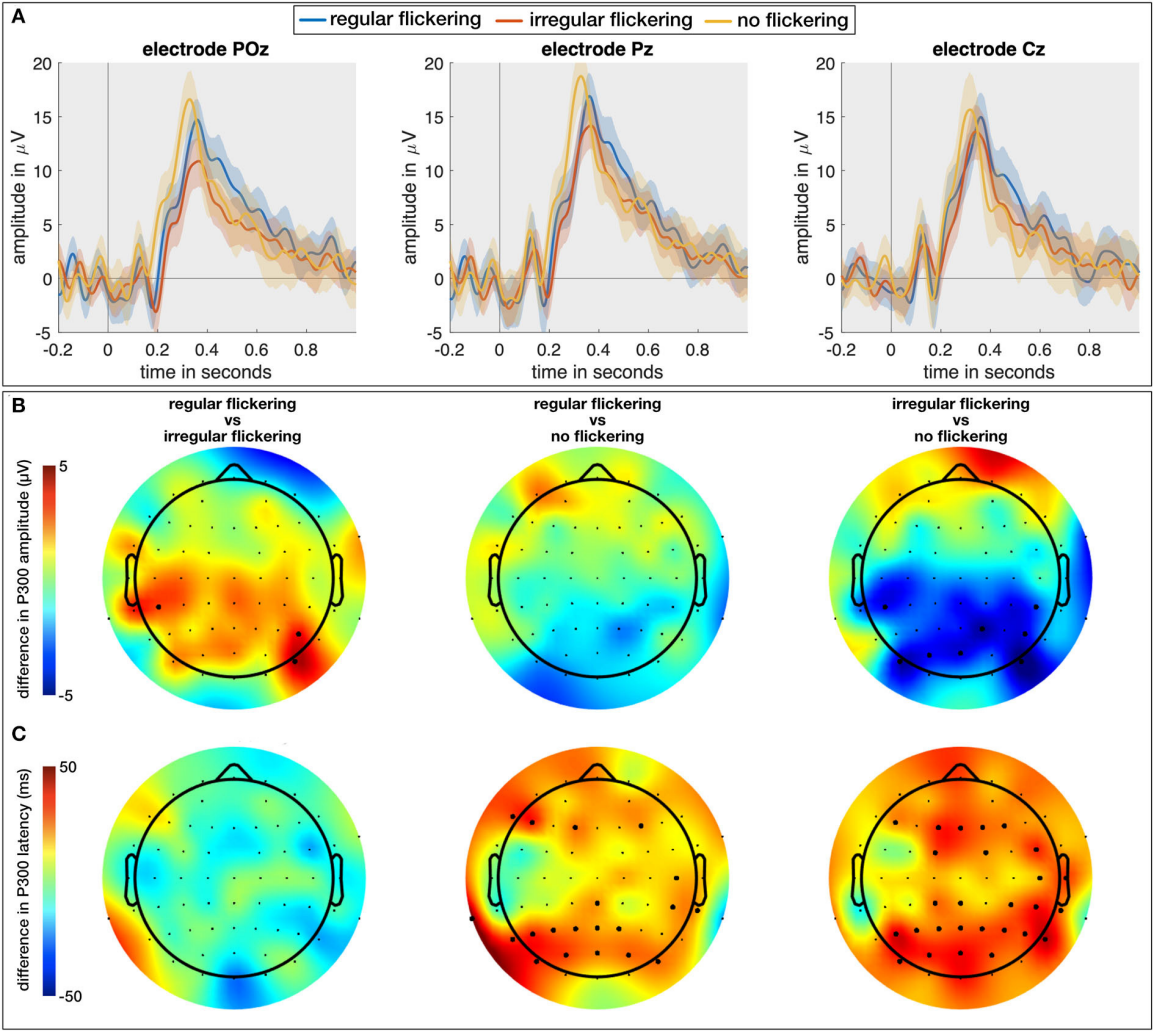
**(A)** The temporal plots (ERPs) in response to regular-oddball, irregular-oddball, and no-flickering-oddball conditions on electrodes POz, Pz, and Cz. Shaded area indicates 95% confidence interval, full-line is the average across the subjects. **(B)** Difference between P300 peak amplitudes [max between 200 and 600 ms (in μV)] between groups accounting for multiple comparisons (three groups). The largest difference was observed between irregular- and no-flicker conditions with no-flicker condition exhibiting the largest P300 amplitude on several electrodes. **(C)** Scalp plots for differences between P300 peak latencies across three groups accounted for multiple comparisons. The P300 response to flickering stimulus (both regular and irregular) was delayed in comparison to no-flickering stimulus. The electrodes indicating a statistically significant difference in **(B,C)** are presented with thick dots.

In a final analysis, in addition to the regular and irregular conditions with a visual attention task (RO and IO, respectively), we included the neural responses to the visual attention task without visual flickering (O). Here, we investigated the effect of flickering (regular and irregular) on the P300 Event-Related Potential (ERP; Soltani and Knight, 2000). As the P300 is one of the most commonly investigated ERP components in the context of memory, attention, and engagement (Datta et al., 2007), investigating its properties when combined with the visual flickering stimulation would allow us to judge effect of the flickering stimulation on the engagement processes in the brain. We expect to obtain a larger P300 response for oddball combined with regular flickering given that it would positively affect the engagement in comparison with oddball stimulation with combined with irregular flickering. One subject from the EEG cohort did not perform the O-condition and was hence excluded from this analysis. For each of the remaining 14 subjects, we extracted the P300 component for the RO-, IO-, and O-conditions as the maximal amplitude and its peak latency of the average response to the oddball stimulus (i.e., the red LED, see methods section) in the time window between 200 and 600 ms. The Kruskal-Wallis test with condition as independent factor showed a significant effect of condition on the P300 amplitude on a limited number of electrodes in the occipito-parieto-central (PO7, PO3, POz, PO8, P2, P6, CP5, CP4, CP6) areas (e.g., for electrode POz, χ^2^ = 10.92, *p* = 0.0043; for electrode P2, χ^2^ = 9.33, *p* = 0.0094, and for electrode CP4, χ^2^ = 6.1, *p* < 0.05) (Figure 6A). A further multiple comparison with the Wilcoxon rank-sum test and FDR correction for multiple comparison shows that this effect is mainly driven by a significantly smaller P300 peak amplitude for the IO condition compared to the O condition, which is consistent for several neighboring parieto-occipital electrodes (e.g., on electrode POz, Z = 3.23, *p* = 0.0037). For the other two comparisons, merely few ( ≤ 3) scattered electrodes exhibited a significant difference, rendering this difference less reliable. Figure 6B shows the scalp distribution of the mentioned effects. The thick dots on the scalp-plots represent the significant difference between the pairs of conditions. The Kruskal-Wallis test showed a significant effect of condition on the P300 peak-latency (Figure 6C) at 31 scalp electrodes (out of 62; e.g., for electrode Pz, χ^2^ = 9.51, *p* = 0.0086; for electrode POz, χ^2^ = 12.76, *p* = 0.0017). Further multiple comparison with the Wilcoxon rank-sum test and FDR correction for multiple comparison confirms the observation that this effect is mainly driven by a significantly shorter latency for the O condition (e.g., for electrode POz, Z = 3.31, *p* = 0.0028 with IO and Z = 2.76, *p* = 0.0086 for RO).

## 3. Discussion

In an effort to contribute to the development of more effective non-pharmacological therapies for AD, the current study aimed at deepen our understanding on how the therapy based on cortical gamma entrainment induced by visual stimulation can be improved. We showed that the inclusion of a simple cognitive task while gazing at a flickering 40 Hz stimulation not only results in an enhanced gamma entrainment but also propagates it to additional neural areas on which it can potentially have a sparing effect. We presented evidence from a scalp-EEG cohort, and furthermore corroborated these results using direct intracranial recordings. The latter additionally showed that in the presence of a cognitive task, the gamma entrainment effect is not only noticeable at the cortical surface but also in deep brain areas such as the hippocampus and insula. Entrainment of these deeper regions is of particular importance since those are believed to be among the first ones to be affected by AD, and therefore serve as primary targets for many AD-therapies (Mu and Gage, 2011). Despite this being an exciting observation, it is important to mention that these results do not imply the observed increased entrainment would have a larger effect on amyloid clearance or cognitive decline *per se*. Further studies are needed to tackle these issues.

One of the striking observations in the current study is that the hippocampus reacted similarly to both visual and non-visual cognitive tasks, while barely reacting to the gamma entrainment stimulation without a task. This could be explained by its involvement in memory processing rather than in processing the visual aspect of the stimulation (Bird and Burgess, 2008), which corroborates the importance of including a task during GENUS therapy and clearly reporting this to be able to aggregate the different reports in a future review study. Even though promising, we need to note that these results were obtained from a single subject. A follow-up study with inclusion of multiple intracranial recordings is required to generalize these results. Furthermore, it is important to remember that the data is obtained from a patient with epilepsy that was implanted with intracranial electrodes for clinical reasons. Thus, the brain response from this patient would not necessarily fully correspond to the response from a general population or patients with AD. Even though this is a serious concern but this is at this point the only way of recording intracranial human data within ethical norms.

It has been previously shown (Park et al., 2020) that visual gamma entrainment therapy can improve amyloid clearance in mouse models of AD, but studies with human participants were inconclusive with regard to the efficacy of the GENUS therapies. Importantly, none of the previous human studies considered the effect of the subject's mental activity during the therapy, which we showed here to have a significant impact. Given this, we recommend that further studies disclose whether the participants were instructed to perform a cognitive task during the therapy, and specify the exact type of task in order to assess their effect on potential amyloid clearance in AD patients. The type of task can be important as also in the present work, we showed that the effect of mental counting seems more restricted to centro-parietal areas for most subjects while the gamma entrainment propagated more frontally during a visual attention task.

The analysis of the oddball data showed that regular flickering does not affect the amplitude of the P300-ERP evoked in response to visual attention task while the irregular flickering considerably decreased it in the parietal region. As the P300 component is assumed to reflect attention and engagement (Datta et al., 2007), this finding suggests that regular flickering does not influence these processes while irregular flickering considerably disrupts them. The increased latency for both regular and irregular flickering conditions suggests that an increased workload for both conditions. As irregular flickering is rarely used in studies, additional studies will be needed to corroborate this hypothesis.

In our previous ECoG study, using flickering stimulation between 10 and 15 Hz (Wittevrongel et al., 2018), we observed steady-state responses at the stimulus frequency that were confined to the primary visual cortex (V1) with no spreading to the associative visual cortex (V2). In contrast, in the current study, we observed a more widespread entrainment in the occipital cortex (i.e., beyond V1), even for the control condition without a cognitive task (as in aforementioned study). This seems to suggest that visual gamma entrainment fundamentally differs from the steady-state responses induced by lower stimulation frequencies, which is in line with theories stating that steady-state responses to higher frequencies have a different cortical distribution compared to those elicited by lower frequencies (Vialatte et al., 2010; Wu, 2016). A recent paper (Cimenser et al., 2021) suggests that the lower frequencies in the gamma range propagate to the larger scalp areas compared to the higher frequencies (including 40 Hz). The authors of this paper however, did not use a task and it is still not clear whether the propagation of the entrainment in combination with a task is different in 40 Hz stimulation from other gamma frequencies. Even though it will be undoubtedly interesting to investigate if such propagation is specific to 40 Hz stimulation or general to higher frequencies, it is out of scope of the current study.

While the current work presents compelling evidence that gamma entrainment benefits from a cognitive task, it remains to be seen whether it also promotes improvements in amyloid clearance in AD patients. If the efficacy of the therapy correlates with the strength/propagation of the entrainment, then the introduction of an additional mental task can help to shorten the currently conducted entrainment sessions in order to reach a predefined desired effect. This, together with a safe (He et al., 2021) and less taxing flickering stimulation procedure that is currently underway (Carstensen et al., 2020; Figueiro and Leggett, 2021), would make the non-invasive and non-pharmacological GENUS therapy more attractive and user-friendly. It is worth mentioning that the therapy if and when available with target the MCI or even earlier stages of disease considering the effect of any experimental disease-modifying therapy in the later stages of AD doomed to a failure due to irreversibility of neuronal damage in this stage. Furthermore, the integration of the stimulation in the daily life (e.g., as a background for ipad) would make the more user-friendly and less tiring.

## 4. Methods and materials

### 4.1. Subjects

Fifteen healthy young volunteers (10 female, ages between 21 and 35 years old, 13 right handed, on average 20 years of education) with normal or corrected-to-normal vision participated in the experiment. The healthy participants did not report any current or previous neurological or psychiatric condition. Additionally, one female patient with refractory epilepsy (24 years old, right handed, 16 years of education) was recruited for this study. Implantation of intracranial EEG (electrocorticography and stereo-EEG electrodes) was done in the scope of the patient's clinical workup with the purpose to locate and further remove the epileptogenic zone. The experiment was conducted while the patient was being monitored at the center for epilepsy monitoring at Gent University Hospital. Before participating, the healthy volunteers and the patient were informed about the purpose of the experiment, the procedure and how the data will be used (GDPR). They all read and signed the informed consent form prior approved by the ethical committees of Leuven (healthy subjects) and Gent (patient) University Hospitals.

### 4.2. Stimulation device

The stimulation device (Figure 1A) consisted of an 8 × 8 LED matrix (FND-588XW4SM00BW35, Forge, UK) controlled by an Arduino Uno microcontroller. The LED matrix was embedded in a white plastic enclosure, to diffuse the light emitted by the LEDs over a surface of 8 × 8 cm, and was suspended by a mounting arm whose height could be adjusted to match the position of the subject. In the center, a small red LED was mounted which was used for oddball stimulation (i.e., the visual attention task, see further) and also served as a fixation point for all sessions.

### 4.3. Experimental procedure

During the experiment, both healthy controls and the patient were sitting at a distance of 55 cm from the stimulation device. Background light was slightly dimmed during the experiment. The subjects experienced a light intensity ranging between 225 and 250 lumen when the stimulation device was on compared to the environmental luminosity (between 125 and 140 lumen) when it was off. In total, six experimental conditions (Figure 1B) were developed: Regular flickering-No task (RN), Regular flickering-Visual attention task (oddball, RO), Regular flickering-Mental counting task (RC), Irregular flickering-No task (IN), Irregular flickering-Visual attention task (oddball, IO), and No flickering-Visual attention task (oddball, O). Fourteen out of 15 healthy participants completed all six experimental conditions, while the remaining participant did not perform the O condition. The patient completed four conditions (no IO and O, due to practical constraints). Each condition was presented once for five consecutive minutes, and the total experiment, including the EEG set-up and breaks, was completed within 60–90 min. In the RN condition, a regular 40 Hz stimulation was presented, which consisted of a square-wave pattern with a 50% duty cycle (Figure 1B). During the IN condition an irregular stimulation pattern was presented that was created by appending single periods of square-wave patterns (50% duty cycle) of random integer frequencies between 30 and 50 Hz. The same luminosity was used for both conditions. Participants were requested to gaze at the fixation point (Figure 1A) and to refrain from thinking about anything specific in order to avoid unintentional cognitive activity. In the RO and IO conditions, the regular and irregular stimulation, respectively, was complemented with an oddball stimulation during which the red fixation LED would light up for 250 ms using a jittered interstimulus interval of 30 ± 10 s (Figure 1B). For both conditions, participants were instructed to mentally count the number of oddball occurrences. During the RC condition, the regular 40 Hz stimulation was presented and participants were asked to gaze at the fixation LED while mentally counting up at their own pace as long as the stimulation was on. Note that, contrary to RO and IO conditions, the fixation LED did not light up in this condition. The purpose of this condition was to determine whether there are differences in visual attention during the execution of two cognitive tasks of similar complexity (i.e., counting up or counting the oddballs) would influence the gamma entrainment and its propagation through the brain. Additionally, for the scalp-EEG cohort only, a pure oddball condition (O) was presented where only the fixation LED would light up for 250 ms with a jittered interstimulus interval of 30 ± 10 s during 5 min while no surrounding flickering was present. Similar to the previous oddball conditions, the subjects were instructed to mentally count the occurrences of the oddball. All conditions were presented in pseudo-randomized order and never more than two of the same type of condition (regular or irregular flickering) were presented consecutively. Each condition was initiated by the researcher pressing a button connected to the microcontroller. In between conditions, the participants were asked to take a short break. For both the healthy subjects and the patient, the attendance to the stimulation was monitored in real-time using an eye-tracker (EyeLink 1000 Plus for healthy participants and a Tobii Pro device for the patient). Stimulus presentation was controlled *via* an Arduino microcontroller (Arduino IDE software, version 1.8.16). For the healthy group, the triggers to EEG, signaling the beginning and end of the flickering stimulation, as well as the beginning and end of the oddball presentation were sent *via* a StimTracker device (Cedric, USA). For the patient, this was done *via* MATLAB, 2017 using the serial cable connecting the laptop that controlled the stimulation and the EEG acquisition device.

### 4.4. Scalp and intracranial EEG data acquisition

#### 4.4.1. Scalp EEG

From the group of healthy subjects, scalp EEG was acquired continuously using 62 active Ag/AgCl electrodes evenly distributed over the scalp at locations following the international 10/20 system. The ground and reference electrodes were placed at AFz and FCz locations, respectively. A drop of conductive gel was applied to each electrode to ensure optimal contact between the electrodes and the subject's scalp. The impedance was kept below 5 kΩ throughout the recordings. EEG data was acquired at a sampling rate of 1 kHz using Synamps RT device (Compumedics Europe, Germany) and stored on a laptop for further analysis.

#### 4.4.2. Intracranial EEG

The patient was implanted with subdural grids covering the right occipital (grid 5 × 2 = 10 contacts), posterior temporal (grid 8 × 4 = 32 contacts) and, anterior and basal temporal (2 strips of 1 × 4 contacts) cortices (Figure 5), and three depth electrodes targeting the hippocampus (6 contacts) and insula (2 × 4 contacts) on the right side. The stereo-EEG contacts in hippocampus had size of 5 mm with 10 mm inter-contact spacing, in insula, they had size of 2 mm with 5 mm inter-contact spacing, the electrodes of the grids and strips had size of 3 mm with a center-to-center distance of 10 mm. In addition, 21 scalp-EEG electrodes were evenly distributed over the scalp at locations following the international 10/20 system. Both intracranial and scalp-EEG were recorded continuously using SD LTM 64 Express devices (Micromed, Italy) operating at a sampling rate of 256 Hz. Reference electrodes were placed on both mastoids. The intracranial electrodes were localized as described in our previous studies (Khachatryan et al., 2019; Hnazaee et al., 2020; Wittevrongel et al., 2020): from the pre-implantation MRI scan of the participants, a cortical reconstruction and volumetric segmentation was performed using the FreeSurfer image analysis suite (version 6.0; Fischl, 2012). The FreeSurfer output was then loaded into Brainstorm (Tadel et al., 2011) and co-registered with a post-implant CT using the SPM12 (Penny et al., 2011) extension. The coordinates of the implanted electrodes were then manually obtained from the artifacts in the CT scan and projected on the cortical surface. The obtained electrode locations were then verified by a neurologist based on the intra-operative images, if available. All cortical visualizations were done using the Brainstorm toolbox (Tadel et al., 2011) and custom Matlab (R2019a) scripts.

### 4.5. Data analysis

#### 4.5.1. Preprocessing

Five-minute epochs were extracted locked to the onset of each condition and a notch filter was applied to remove the 50 Hz interference from the powerline.

#### 4.5.2. Power spectrum

The frequency spectrum at each (scalp and intracranial) EEG channel in response to each experimental condition was extracted by cutting the 5 min epoch into 50% overlapping 30 s segments, obtaining the Fourier transform of each segment, and averaging their spectra. The described procedure results in a frequency resolution of 0.033 Hz.

#### 4.5.3. Signal-to-noise ratio

To quantify the extent of the gamma entrainment relative to the background activity, the experimental conditions were compared using the signal-to-noise ratio (SNR) of the neural response at 40 Hz. The SNR was obtained as the ratio of the (Fourier) amplitude at 40 Hz and the average of the amplitudes in the range from 38 to 42 Hz, excluding 40 Hz. Note that a SNR equal to 1 indicates that the 40 Hz amplitude does not stand out from the background activity.

#### 4.5.4. Oddball analysis

The scalp-EEG data from the oddball conditions was offline re-referenced to the average of the mastoid electrodes (TP9 and TP10) and filtered between 0.5 and 15 Hz using a 4th order zero-phase Butterworth filter. Next, the EEG recording was cut into epochs starting from 200 ms before until 1,000 ms after the onset of each oddball stimulus. The epochs were baselined to the average of the 200 ms pre-onset signal. The epochs for which the maximal amplitude exceeded ±100 μV were rejected as they were considered artifactual. As a measure of the P300 event related potentials (ERP) we took the maximal positive amplitude between 200 and 600 ms post-onset (i.e., peak amplitude) as well as its latency relative to the stimulus onset.

#### 4.5.5. Eye-tracking data

The subjects' gaze during the sessions was monitored using eye-tracking of the left eye. For each session, we first cleaned the artifacts in the eye-tracking signal that originated from eye blinks. Eye blinks were determined online by the Eyelink software and stored as timestamped messages in the offline data file. We then determined the standard deviation of the remaining gaze signal (i.e., after eye blink removal) in both horizontal and vertical axis.

### 4.6. Statistics

For the descriptive statistics, we used the median SNR across subjects and calculated its 95% confidence interval (CI) using a bootstrapping procedure with 1,000 samples selected with replacement. In order to eliminate the effect of outliers on the obtained results we opted for non-parametric statistical testing on SNR evaluation, which, even though less powerful, is less prone to the biases of outliers. The SNRs, initially across all five conditions (RN, RO, RC, IN, and IO) and afterwards across the regular conditions (RN, RO, and RC) separately, were compared using the non-parametric Kruskal-Wallis test, where experimental conditions served as independent variable and SNR on each channel as dependent variables. Then, a multiple comparison across the regular conditions with the same Kruskal-Wallis test was performed, using the false discovery rate (FDR) correction (Benjamini Yoav, 1995) for multiple comparison. The spread of activation for SNRs for each subject was compared across experimental conditions using a cluster-based permutation test (Maris and Oostenveld, 2007) using the Matlab-based Fieldtrip toolbox (Oostenveld et al., 2011) to find spatial clusters that exhibit a significant difference in response to stimulation groups. We used a Monte-Carlo method to perform a significance probability mapping using the one-tailed independent *t*-test and max-sum as cluster statistics. At least two neighboring electrodes had to pass the significance threshold of 0.05 for it to be considered a cluster. The number of permutations was set to 2,000. The significance threshold was set to 0.05 for all conditions and subjects. The effect of the type of flickering (i.e., regular, irregular or no flickering) on the P300-ERP was assessed using a Kruskal-Wallis non-parametric test with condition (O, RO, and IO) as independent variable and peak amplitude/latency of the P300 on each electrode as a dependent variable. Further multiple comparison between these conditions was done using the same Kruskal-Wallis test with FDR-correction for *p*-values. The effect of condition (four conditions) on the intracranial data was assessed using the Kruskal-Wallis non-parametric test, and further multiple comparison was performed using a non-parametric Wilcoxon rank-sum test with FDR-corrections for multiple comparisons. For all analyses, the threshold for statistical significance was kept at 0.05.

## Data availability statement

The datasets presented in this article are not readily available because of the sensitive nature of intracranial patient data. Requests to access the datasets should be directed to corresponding author: elvira.khachatryan@ugent.be for scalp-EEG data and to EC, evelien.carrette@uzgent.be for intracranial data.

## Ethics statement

The studies involving human participants were reviewed and approved by Ethische Commissie Onderzoek, UZ Leuven and Commissie voor Medische Ethiek, UZ Gent. The patients/participants provided their written informed consent to participate in this study.

## Author contributions

EK, BW, and MV conceptualized the study. EK and BW developed the paradigm and analyzed the data. BW implemented the paradigm. EK, MR, EC, PB, AM, ID, and DV recruited the participants and collected the data. All authors participated in writing the manuscript. All authors contributed to the article and approved the submitted version.

## Funding

The work has been performed while EK and BW were employed by KU Leuven. MV was supported by research grants received from the European Union's Horizon 2020 research and innovation programme under grant agreement No. 857375, the special research fund of the KU Leuven (C24/18/098), the Belgian Fund for Scientific Research—Flanders (G088314N, G0A0914N, G0A4118N, and G0A4321N), the Inter-university Attraction Poles Programme—Belgian Science Policy (IUAP P7/11), and the Hercules Foundation (AKUL 043).

## Conflict of interest

The authors declare that the research was conducted in the absence of any commercial or financial relationships that could be construed as a potential conflict of interest.

## Publisher's note

All claims expressed in this article are solely those of the authors and do not necessarily represent those of their affiliated organizations, or those of the publisher, the editors and the reviewers. Any product that may be evaluated in this article, or claim that may be made by its manufacturer, is not guaranteed or endorsed by the publisher.

## References

[B1] Adaikkan C. Middleton S. J. Marco A. Pao P.-C. Mathys H. Kim D. N.-W.. (2019). Gamma entrainment binds higher-order brain regions and offers neuroprotection. Neuron 102, 929–943. 10.1016/j.neuron.2019.04.011 31076275 PMC6697125

[B2] Adaikkan C. Tsai L.-H. (2020). Gamma entrainment: impact on neurocircuits, glia, and therapeutic opportunities. Trends Neurosci. 43, 24–41. 10.1016/j.tins.2019.11.001 31836315

[B3] Alzheimer's Association (2020). 2020 Alzheimer's disease facts and figures. Alzheimers Dement. 16, 391–460. 10.1002/alz.12068 32157811

[B4] Benjamini Yoav H. Y. (1995). Controlling the false discovery rate: a practical and powerful approach to multiple testing. J. R. Stat. Soc. 57, 289–300. 10.1111/j.2517-6161.1995.tb02031.x

[B5] Bird C. M. Burgess N. (2008). The hippocampus and memory: insights from spatial processing. Nat. Rev. Neurosci. 9, 182–194. 10.1038/nrn2335 18270514

[B6] Breijyeh Z. Karaman R. (2020). Comprehensive review on Alzheimer's disease: causes and treatment. Molecules 25:5789. 10.3390/molecules25245789 33302541 PMC7764106

[B7] Carstensen M. S. Lindén J. Nguyen N. M. Hansen H. E. Carrillo G. M. F. Hansen L. S.. (2020). “40 Hz invisible spectral flicker and its potential use in Alzheimer's light therapy treatment,” in Mechanisms of Photobiomodulation Therapy XV, Vol. 11221, San Francisco, 47–58. 10.1117/12.2544338

[B8] Chan D. Suk H.-J. Jackson B. Milman N. P. Stark D. Klerman E. B.. (2021). 40 Hz sensory stimulation induces gamma entrainment and affects brain structure, sleep and cognition in patients with Alzheimer's dementia. *medRxiv*. 1–3. 10.1101/2021.03.01.21252717

[B9] Cimenser A. Hempel E. Travers T. Strozewski N. Martin K. Malchano Z.. (2021). Sensory-evoked 40-hz gamma oscillation improves sleep and daily living activities in Alzheimer's disease patients. Front. Syst. Neurosci. 15:746859. 10.3389/fnsys.2021.746859 34630050 PMC8500065

[B10] Datta A. Cusack R. Hawkins K. Heutink J. Rorden C. Robertson I. H.. (2007). The p300 as a marker of waning attention and error propensity. Comput. Intell. Neurosci. 2007:93968. 10.1155/2007/93968 18301718 PMC2246084

[B11] Figueiro M. G. Leggett S. (2021). Intermittent light exposures in humans: a case for dual entrainment in the treatment of Alzheimer's disease. Front. Neurol. 12:625698. 10.3389/fneur.2021.625698 33767659 PMC7985540

[B12] Fischl B. (2012). Freesurfer. Neuroimage 62, 774–781. 10.1016/j.neuroimage.2012.01.021 22248573 PMC3685476

[B13] Guan A. Wang S. Huang A. Qiu C. Li Y. Li X.. (2022). The role of gamma oscillations in central nervous system diseases: mechanism and treatment. Front. Cell. Neurosci. 16:962957. 10.3389/fncel.2022.962957 35966207 PMC9374274

[B14] He Q. Colon-Motas K. M. Pybus A. F. Piendel L. Seppa J. K. Walker M. L.. (2021). A feasibility trial of gamma sensory flicker for patients with prodromal Alzheimer's disease. Alzheimer's Dementia 7:e12178. 10.1002/trc2.12178 34027028 PMC8118113

[B15] Hebert L. E. Weuve J. Scherr P. A. Evans D. A. (2013). Alzheimer disease in the United States (2010-2050) estimated using the 2010 census. Neurology 80, 1778–1783. 10.1212/WNL.0b013e31828726f5 23390181 PMC3719424

[B16] Hnazaee M. F. Wittevrongel B. Khachatryan E. Libert A. Carrette E. Dauwe I.. (2020). Localization of deep brain activity with scalp and subdural EEG. NeuroImage 223:117344. 10.1016/j.neuroimage.2020.117344 32898677

[B17] Iaccarino H. F. Singer A. C. Martorell A. J. Rudenko A. Gao F. Gillingham T. Z.. (2016). Gamma frequency entrainment attenuates amyloid load and modifies microglia. Nature 540, 230–235. 10.1038/nature20587 27929004 PMC5656389

[B18] Ismail R. Hansen A. K. Parbo P. Brændgaard H. Gottrup H. Brooks D. J.. (2018). The effect of 40-hz light therapy on amyloid load in patients with prodromal and clinical Alzheimer's disease. Int. J. Alzheimer's Dis. 2018:6852303. 10.1155/2018/6852303 30155285 PMC6091362

[B19] Jack Jr C. R. Bennett D. A. Blennow K. Carrillo M. C. Dunn B. Haeberlein S. B.. (2018). Nia-AA research framework: toward a biological definition of Alzheimer's disease. Alzheimer's Dement. 14, 535–562. 10.1016/j.jalz.2018.02.018 29653606 PMC5958625

[B20] Khachatryan E. Wittevrongel B. Hnazaee M. F. Carrette E. Dauwe I. Meurs A.. (2019). Semantic and perceptual priming activate partially overlapping brain networks as revealed by direct cortical recordings in humans. NeuroImage 203:116204. 10.1016/j.neuroimage.2019.116204 31539593

[B21] Knopman D. S. Amieva H. Petersen R. C. Chételat G. Holtzman D. M. Hyman B. T.. (2021). Alzheimer disease. Nat. Rev. Dis. Primers, 7, 1–21. 10.1038/s41572-021-00269-y 33986301 PMC8574196

[B22] Maris E. Oostenveld R. (2007). Nonparametric statistical testing of EEG-and MEG-data. J. Neurosci. Methods 164, 177–190. 10.1016/j.jneumeth.2007.03.024 17517438

[B23] Martorell A. J. Paulson A. L. Suk H.-J. Abdurrob F. Drummond G. T. Guan W.. (2019). Multi-sensory gamma stimulation ameliorates Alzheimer's-associated pathology and improves cognition. Cell 177, 256–271. 10.1016/j.cell.2019.02.014 30879788 PMC6774262

[B24] MATLAB (2017). Version 2017b. Natick, MA: The MathWorks Inc.

[B25] Mu Y. Gage F. H. (2011). Adult hippocampal neurogenesis and its role in Alzheimer's disease. Mol. Neurodegener. 6, 1–9. 10.1186/1750-1326-6-85 22192775 PMC3261815

[B26] Mullard A. (2021). Controversial Alzheimer's drug approval could affect other diseases. Nature 595, 162–163. 10.1038/d41586-021-01763-9 34193994

[B27] Oostenveld R. Fries P. Maris E. Schoffelen J.-M. (2011). Fieldtrip: open source software for advanced analysis of MEG, EEG, and invasive electrophysiological data. Comput. Intell. Neurosci. 2011:156869. 10.1155/2011/156869 21253357 PMC3021840

[B28] Park S.-S. Park H.-S. Kim C.-J. Kang H.-S. Kim D.-H. Baek S.-S.. (2020). Physical exercise during exposure to 40-hz light flicker improves cognitivefunctions in the 3XTG mouse model of Alzheimer's disease. Alzheimer's Res. Ther. 12, 1–15. 10.1186/s13195-020-00631-4 32434556 PMC7240923

[B29] Penny W. D. Friston K. J. Ashburner J. T. Kiebel S. J. Nichols T. E. (2011). Statistical Parametric Mapping: The Analysis of Functional Brain Images. London: Elsevier.

[B30] Sharpe R. L. Mahmud M. Kaiser M. S. Chen J. (2020). Gamma entrainment frequency affects mood, memory and cognition: an exploratory pilot study. Brain Informatics 7, 1–12. 10.1186/s40708-020-00119-9 33226543 PMC7683678

[B31] Silberstein R. B. Nunez P. L. Pipingas A. Harris P. Danieli F. (2001). Steady state visually evoked potential (SSVEP) topography in a graded working memory task. Int. J. Psychophysiol. 42, 219–232. 10.1016/S0167-8760(01)00167-2 11587778

[B32] Soltani M. Knight R. T. (2000). Neural origins of the p300. Crit. Rev. Neurobiol. 14, 199–224. 10.1615/CritRevNeurobiol.v14.i3-4.20 12645958

[B33] Sperling R. A. Aisen P. S. Beckett L. A. Bennett D. A. Craft S. Fagan A. M.. (2011). Toward defining the preclinical stages of Alzheimer's disease: recommendations from the national institute on aging-Alzheimer's association workgroups on diagnostic guidelines for Alzheimer's disease. Alzheimer's Dement. 7, 280–292. 10.1016/j.jalz.2011.03.003 21514248 PMC3220946

[B34] Tadel F. Baillet S. Mosher J. C. Pantazis D. Leahy R. M. (2011). Brainstorm: a user-friendly application for MEG/EEG analysis. Comput. Intell. Neurosci. 2011:879716. 10.1155/2011/879716 21584256 PMC3090754

[B35] Toffanin P. de Jong R. Johnson A. Martens S. (2009). Using frequency tagging to quantify attentional deployment in a visual divided attention task. Int. J. Psychophysiol. 72, 289–298. 10.1016/j.ijpsycho.2009.01.006 19452603

[B36] Vialatte F.-B. Maurice M. Dauwels J. Cichocki A. (2010). Steady-state visually evoked potentials: focus on essential paradigms and future perspectives. Prog. Neurobiol. 90, 418–438. 10.1016/j.pneurobio.2009.11.005 19963032

[B37] Wittevrongel B. Khachatryan E. Carrette E. Boon P. Meurs A. Van Roost D.. (2020). High-gamma oscillations precede visual steady-state responses: a human electrocorticography study. Hum. Brain Mapp. 41, 5341–5355. 10.1002/hbm.25196 32885895 PMC7670637

[B38] Wittevrongel B. Khachatryan E. Hnazaee M. F. Carrette E. De Taeye L. Meurs A.. (2018). Representation of steady-state visual evoked potentials elicited by luminance flicker in human occipital cortex: an electrocorticography study. Neuroimage 175, 315–326. 10.1016/j.neuroimage.2018.04.006 29630994

[B39] Wu Z. (2016). Physical connections between different SSVEP neural networks. Sci. Rep. 6, 1–9. 10.1038/srep22801 26952961 PMC4782133

