# Supplementary material for: Cognitive tasks propagate the neural entrainment in response to a visual 40 Hz stimulation in humans
Source: Front Aging Neurosci. 2022 Oct 6;14:1010765. doi: 10.3389/fnagi.2022.1010765 (PMC9582357; doi:10.3389/fnagi.2022.1010765)
Supplement: Supplementary file 1 [file Data_Sheet_1.PDF]

Supplementary Material

1 SUPPLEMENTARY FIGURES

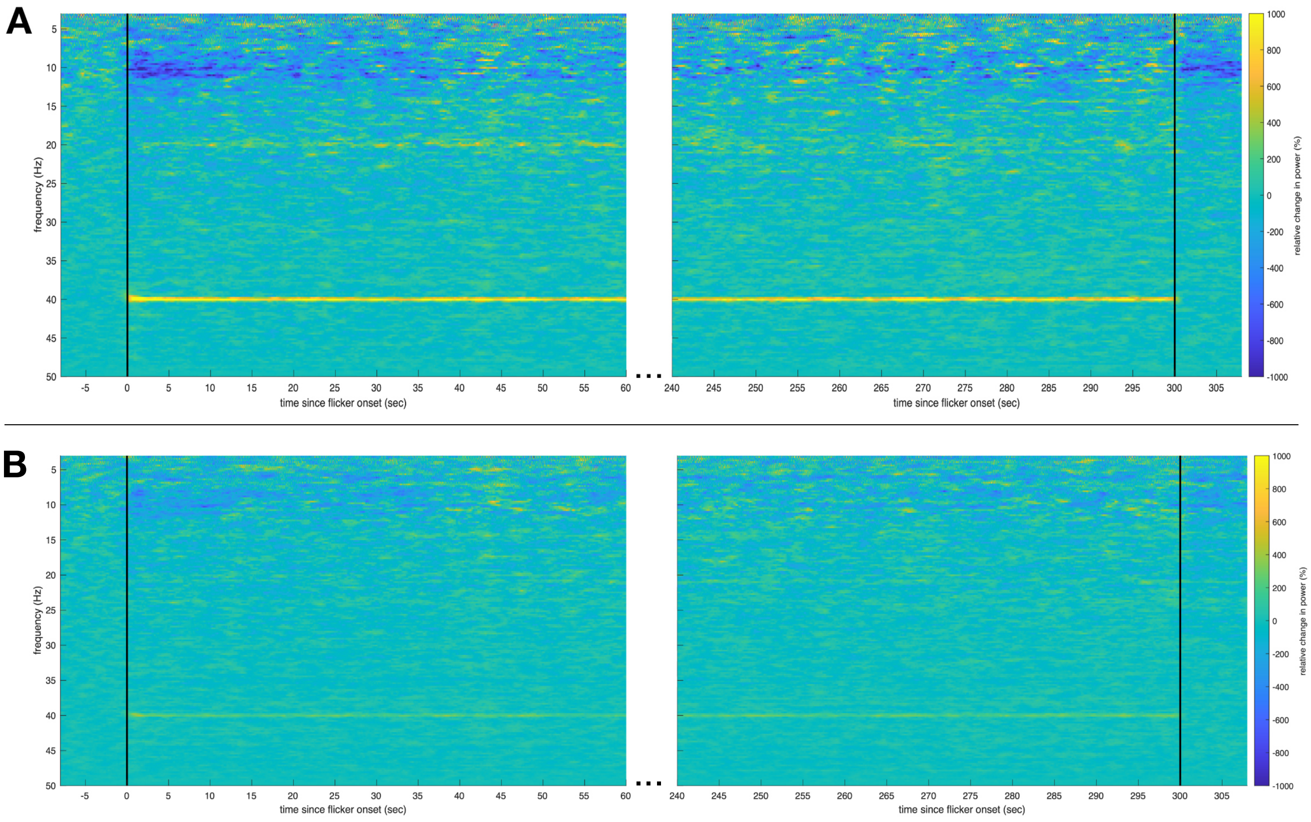

Figure S1. Time frequency analysis of the no-task condition with regular flickering at channel (A) Oz and (B) CPz

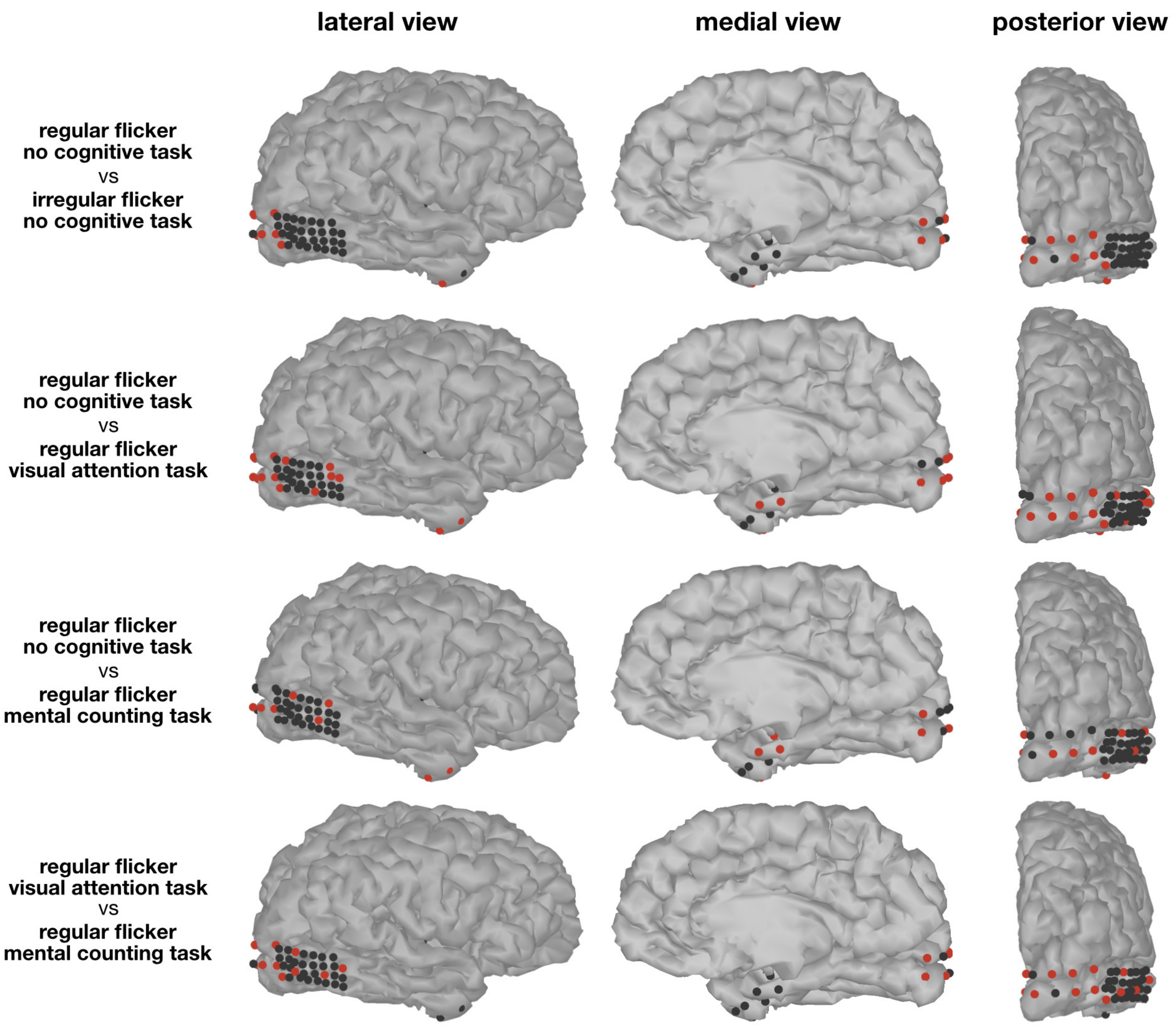

Figure S2. The results of multiple comparison on individual electrode of ECoG grids. The electrodes colored in red show significance for the comparison specified on the left side of the figure.
